# A longitudinal study of cerebral blood flow under hypoxia at high altitude using 3D pseudo-continuous arterial spin labeling

**DOI:** 10.1038/srep43246

**Published:** 2017-02-27

**Authors:** Wenjia Liu, Jie Liu, Xin Lou, Dandan Zheng, Bing Wu, Danny J. J. Wang, Lin Ma

**Affiliations:** 1 Department of Radiology, Chinese PLA General Hospital, Beijing, China; 2 Department of Radiology, Tibet Military General Hospital, Lhasa, Tibet, China; 3 Department of Neurology, University of California, Los Angeles, CA, USA; 4 GE Healthcare, MR Research China, Beijing, China

## Abstract

Changes in cerebral blood flow (CBF) may occur with acute exposure to high altitude; however, the CBF of the brain parenchyma has not been studied to date. In this study, identical magnetic resonance scans using arterial spin labeling (ASL) were performed to study the haemodynamic changes at both sea level and high altitude. We found that with acute exposure to high altitude, the CBF in acute mountain sickness (AMS) subjects was higher (P < 0.05), while the CBF of non-AMS subjects was lower (P > 0.05) compared with those at sea level. Moreover, magnetic resonance angiography in both AMS and non-AMS subjects showed a significant increase in the cross-sectional areas of the internal carotid, basilar, and middle cerebral arteries on the first day at high altitude. These findings support that AMS may be related to increased CBF rather than vasodilation; these results contradict most previous studies that reported no relationship between CBF changes and the occurrence of AMS. This discrepancy may be attributed to the use of ASL for CBF measurement at both sea level and high altitude in this study, which has substantial advantages over transcranial Doppler for the assessment of CBF.

Hypobaric hypoxia occurs with acute exposure to high altitude, which may result in acute mountain sickness (AMS). AMS is characterized by symptoms such as headache, nausea, vomiting, fatigue, dizziness and sleeping disorders^1^. However, the exact underlying physiological mechanism remains unknown. Furthermore, hypoxia is concomitant with other diseases such as ischemic stroke and epilepsy^2^, hence the study of cerebrovascular response to hypoxia is of great clinical significance. Baumgartner *et al*.^3^ first reported increased cerebral blood flow (CBF) in those with AMS compared to those without. However, this was not observed in later studies^4^,^5^,^6^,^7^,^8^,^9^. The role of CBF in the pathogenesis of AMS remains a subject of debate.

The inconsistency of previous results might be attributed to methodology or study design. Previous studies either used transcranial Doppler (TCD)^10^,^11^,^12^,^13^,^14^,^15^,^16^,^17^,^18^ or simulated hypoxia at sea level^11^,^19^,^20^; both approaches have certain substantial limitations. While TCD has the benefit of portability, it only offers measures of arterial flow velocity with or without middle cerebral artery (MCA) diameters, rather than actual CBF^14^. The assessment of CBF using TCD relies on the key assumptions that the arterial diameter remains unchanged and that the arterial blood flow is frictionless laminar flow^10^. However, both assumptions might be violated in reality. Wilson *et al*.^11^ reported that the MCA diameter increased at high altitude, and the blood flow is consequently no longer laminar. Furthermore, CBF was calculated using fixed diameter and velocity with TCD, but true CBF is derived using the integral of the blood velocity across different arterial sections throughout the entire cardiac cycle. Moreover, the reproducibility of TCD is questionable as changes in the insonation angle and subject movement may lead to variations in TCD measurements^21^. Simulated hypoxia can only replicate the short-term effects of hypoxia, which differ from the actual high altitude environment in which long-term exposure to hypoxia occurs. In addition, the environmental variations at high altitude compared to sea level are more complicated than hypoxia alone. Therefore, the observations made in the simulated hypoxia environment may not accurately reflect the changes that occur at high altitude.

In this study, 3D pseudo-continuous arterial spin labeling (3D pCASL) at 3.0 T is used to investigate the CBF changes at both sea level and high altitude. Longitudinal measurements of CBF values and cross-sectional areas of major cerebral arteries were made at sea level, after acute exposure to high altitude, after acclimatization at high altitude, and after returning to sea level. To minimize the bias in measurement, identical MR scanners, receiver coils, scan protocols, as well as post processing were applied. AMS and non-AMS subjects were identified at high altitude, and analyses were performed to assess the cerebrovascular responses to hypoxia.

## Results

### Clinical tests and haematology

The clinical and haematology data obtained are shown in Table 1. No change in mean arterial pressure (MAP) from sea level to high altitude was observed in AMS or non-AMS subjects. Arterial oxygen saturation (SaO_2_) dropped from 98.40 ± 0.16 (SL1) to 86.00 ± 1.24 (HA1) (P < 0.01) after acute exposure to high altitude and recovered to 98.30 ± 0.15 (SL2) upon return to sea level. Heart rate (HR) increased from 69.30 ± 2.46 (SL1) to 78.40 ± 3.85 (HA1) (P < 0.01). Hematocrit (Hct) increased from 0.402 ± 0.010 (SL1) to 0.440 ± 0.010 (HA2) (P < 0.05) after acclimatization at high altitude, while hemoglobin (Hb) increased from 136.40 ± 3.81 (SL1) to 149.30 ± 4.38 (HA2) (P < 0.05). Seven subjects in the cohort were in the AMS group (LLS score: 4.71 ± 2.14 points) and 3 subjects were in the non-AMS group (LLS score: 1.67 ± 0.58 points) on day 3. However, on day 6 (HA2), the AMS symptoms disappeared in the AMS group with acclimatization at high altitude (LLS score: 0.88 ± 0.30).

### Cerebral blood flow

**Figure 1:**
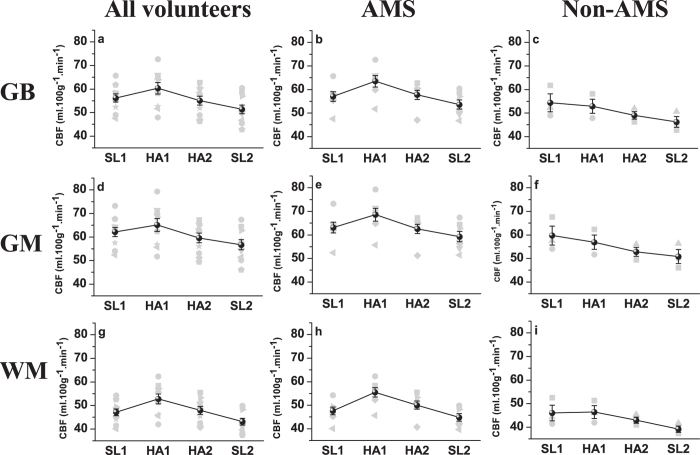
Individual (grey) and mean (black) cerebral blood flow (CBF) at sea level (SL1), the first day at high altitude (HA1), the last day at high altitude (HA2) and returning to sea level (SL2). (**a**,**b**,**c**) are CBF in global brain (GB); (**d**,**e**,**f**) are CBF in grey matter (GM); and (**g**,**h**,**i**) are CBF in white matter (WM). Error bar: sem.

The CBF measurements obtained using a post-labeling delay (PLD) of 1.5 s, 2.0 s, and 2.5 s showed similar trends, the results with a PLD of 2 s were used for analysis (Fig. 1).

**Figure 2:**
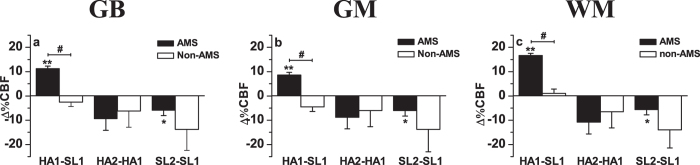
Cerebral blood flow (CBF) changes of AMS and non-AMS subjects in global brain (GB) (**a**), grey matter (GM) (**b**), and white matter (WM) (**c**). HA1-SL1: change in CBF between HA1 and SL1; HA2-HA1: change in CBF between HA2 and HA1; SL2-SL1: change in CBF between SL2 and SL1. CBF between HA1 and SL1, HA2 and HA1, SL2 and SL1 in AMS subjects were compared using paired samples t-test, whereas related samples nonparametric test was used in non-AMS subjects; comparison between AMS and non-AMS subjects used the independent samples nonparametric test. Data are mean changes. Error bar: sem. *P < 0.05; **P < 0.01; ^#^P < 0.05.

In all subjects, the global brain (GB) CBF value increased from 56.22 ± 1.75 (SL1) to 60.32 ± 2.45 (HA1) (P < 0.05), while the CBF value decreased to 55.11 ± 1.90 (HA2) after acclimatization at high altitude (P < 0.05, compared with HA1), and further dropped to 51.39 ± 1.84 (SL2) after returning to sea level (P < 0.05, compared with SL1) (Fig. 1). Different trends in CBF variations were observed between AMS and non-AMS subjects after exposure to high altitude; the GB CBF in AMS subjects increased from 57.02 ± 2.03 (SL1) to 63.52 ± 2.43 (HA1) (P < 0.01), while that of non-AMS subjects decreased from 54.34 ± 3.81 (SL1) to 52.88 ± 2.99 (HA1) (P > 0.05). The difference in GB CBF changes with exposure to high altitude (HA1—SL1) was statistically significant (P < 0.05) between AMS and non-AMS subjects (Fig. 2).

The grey matter (GM) and white matter (WM) CBF values of all subjects increased from SL1 to HA1 (P < 0.05), then decreased after acclimatization at high altitude (HA2) (P < 0.05, compared with HA1) and further dropped after altitude (SL2) (P < 0.05, compared with SL1) (Fig. 1). The CBF changes in GM and WM with exposure to high altitude (HA1—SL1) were significantly different (P < 0.05) between AMS and non-AMS subjects (Fig. 2). With acute exposure to high altitude (HA1), both GM and WM CBF values in AMS subjects increased compared with those at sea level (SL1), and the percentage of CBF increase was greater in WM than in GM. However, the CBF values of both GM and WM in non-AMS subjects showed no evident changes between HA1 and SL1 (Fig. 2).

### Cross-sectional areas of arteries

**Figure 3:**
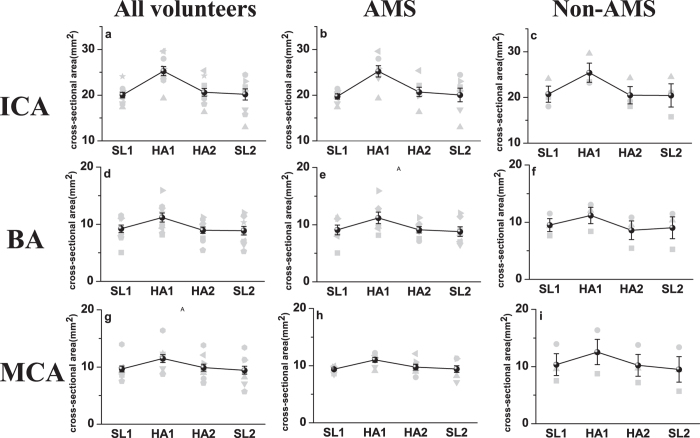
Individual (grey) and mean (black) cross-sectional area at sea level (SL1), the first day at high altitude (HA1), the last day at high altitude (HA2) and returning to sea level (SL2). (**a**,**b**,**c**) are cross-sectional areas of internal carotid (ICA); (**d**,**e**,**f**) are cross-sectional areas of basilar artery (BA); and (**g**,**h**,**i**) are cross-sectional areas of middle cerebral artery (MCA). Error bar: sem.

**Figure 4:**
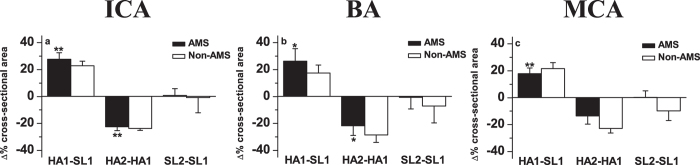
Cross-sectional area changes of AMS and non-AMS subjects in internal carotid (ICA) (**a**), basilar artery (BA) (**b**), and middle cerebral artery (MCA) (**c**). HA1-SL1: change in cross-sectional area between HA1 and SL1; HA2-HA1: change in cross-sectional area between HA2 and HA1; SL2-SL1: change in cross-sectional area between SL2 and SL1. Cross-sectional areas between HA1 and SL1, HA2 and HA1, SL2 and SL1 in AMS subjects were compared using paired samples t-test, whereas related samples nonparametric test was used in non-AMS subjects; comparison between AMS and non-AMS subjects used the independent samples nonparametric test. Data are mean changes. Error bar: sem. *P < 0.05; **P < 0.01.

In all subjects, including AMS and non-AMS, the cross-sectional areas of internal carotid artery (ICA), basilar artery (BA), and middle cerebral artery (MCA) showed an increase at HA1 (P < 0.05, compared with SL1), the values then returned after acclimatization to high altitude (HA2) and after altitude (SL2) (P > 0.05, compared with SL1) (Fig. 3). There was no significant difference between the measurements in AMS and non-AMS subjects (Fig. 4).

## Discussion

A distinctive advantage of this study is the direct acquisition of CBF values and cross-sectional areas of major intracranial arteries with 3D pCASL and MRA at high altitude. Identical MR scanners, receiver coils, and pulse sequences were used for data acquisition to eliminate potential bias. The directly-measured CBF values using ASL were in absolute units and reflected the micro-circulation in brain tissues. Region-specific and tissue-specific measurements could be obtained because of 3D whole brain acquisition. CBF measurements using ASL were found to be reproducible in multi-center studies^22^,^23^. In contrast, the approaches based on either TCD or simulated hypoxia may have some limitations: (1) the cross-sectional areas of major arteries obviously varied when moving from sea level to high altitude, which disrupted the assumption of TCD; and (2) the Hct measurement showed that the haematological conditions featured a middle-to-long term alternation that could not be observed in a short-term simulated hypoxia environment.

The measured MAP values did not change after acute exposure to high altitude on day 2 compared to those obtained at sea level; no difference was observed between the AMS and non-AMS subjects. This observation suggested that the changes in CBF were not strongly correlated with the changes in MAP. It is well known that oxygen pressure decreases with elevated altitude, leading to hypobaric hypoxia, and the SaO_2_ was observed to drop at high altitude and returned to normal upon return to sea level in normoxia. Hct and Hb also increased after acclimatization to hypoxia at high altitude, which may be associated with the decrease in CBF after acclimatization to hypoxia at high altitude.

The CBF of AMS subjects showed an increase after ascending to high altitude on day 2 compared to at sea level, whereas the CBF of non-AMS subjects did not show obvious changes. This finding in contrast with most previous studies in which no relationship was observed between changes in CBF and the severity of AMS symptoms at high altitude^1^,^9^,^24^,^25^, except for those of Baumgartner *et al*.^3^ and Feddersen *et al*.^12^. This could be because the previous studies relied on the blood velocity measurement in major arteries by TCD for the assessment of CBF, which could be unreliable as previously discussed. On the other hand, the cross-sectional areas of the ICA, BA and MCA were found to increase in both AMS and non-AMS subjects after acute exposure to hypoxia at high altitude. Our finding is consistent with several previous reports. Willie *et al*.^26^ found that the ICA showed an approximately 20% increase in luminal diameter through a PaCO2 range of 15–65 mmHg, while Wilson *et al*.^11^ found an increase in the MCA diameter with exposure to extreme hypobaric hypoxia.

At high altitude with hypoxia, ventilation is improved to maintain the oxygen content in the blood as a form of body regularization, which consequently leads to a decreased level of CO_2_ in the blood^27^. However, the low oxygen level as a result of hypoxia and decreased CO2 (known as hypocapnia) lead to artery vasodilation and vasoconstriction respectively^28^, hence the change of arterial dimension with exposure to hypoxia depends on the relative strengths of the two simultaneous and opposing effects. Based on our and others’ observation that the cross-sectional areas of major arteries were enlarged, the hypoxia caused vasodilation was a more dominant factor for large arteries (such as ICA, BA, and MCA) with acute exposure to high altitude. However, instead of the dimension changes of large arteries, the dimensions of arterioles have more determinant impacts on the resulting CBF. The arteriole dimensions are not only affected by the level of blood gases (oxygen and CO2 content in the blood), but also largely impacted by metabolic changes^29^,^30^. A distinction of arterioles from large arteries (such as ICA, BA, and MCA) is that they are located in the subarachnoid space, tethered to the abutting mater and surrounded by CSF. As a result, the regulation of arterioles is also affected by the local CSF environment that is determined by metabolic conditions. The detailed cerebrovascular physiology governing arterioles remains unclear due to difficulties in methodology. Given variations in CBF and in changes of arterial dimensions observed in AMS and non-AMS subjects, it may be postulated that the main factor that contributes to the CBF difference between the two groups is the regulation of arterioles.

High altitude cerebral edema (HACE) might occur if AMS develops^31^. Previous studies have shown that the edema is primarily localized in the WM^32^,^33^. The present study found that the percentage of CBF increase with acute exposure to high altitude was greater in WM than in GM in AMS subjects. The disturbance of normal CBF in WM might partially explain the mechanism of HACE.

The CBF in the GB, GM and WM of all subjects decreased to sea level values on day 6 at high altitude after acclimatization. No difference was observed between AMS and non-AMS subjects. Moreover, the AMS symptoms disappeared in AMS subjects on day 6 at high altitude. The cross-sectional areas of ICA, BA and MCA also returned to sea level values on day 6 at high altitude for both AMS and non-AMS subjects. Acclimatization to hypoxia at high altitude may take days to months^30^,^34^. In this process, the oxygen content is progressively restored and the vasodilation effects gradually vanish so that the cross-sectional areas of the arteries return to the sea level value as observed in this study. Previous studies have also shown that ventilatory acclimatization coincides with the fall in CBF at high altitude due to an increase in oxygen content and decrease in CO2 resulting from reflex hyperventilation^35^,^36^,^37^. The drop of CBF compared to initial exposure to hypoxia was also supported by the elevated Hct and Hb levels on day 6 at high altitude, which is known to have an inverse relationship with CBF^25^.

The GB, GM and WM CBF in all subjects further decreased after returning to sea level in a normoxic environment and were even lower than the values obtained before ascending to high altitude. These findings contradicted the results of Villien *et al*.^10^, which showed an increase in CBF within 6 hours after returning to sea level. The inconsistency results may be caused by the different time periods for returning to sea level. Participants in the study of Villien *et al*.^10^ were transported back to sea level by helicopter (rapid descent), while participants in our study returned back to seal level via 2-day train ride (gradual descent). In our study, we speculate that the elevated Hct as well as hypocapnia, compared to the original physiological condition at sea level, would maintain a low CBF level. We know that the decrease in Hct is a middle-to-long term process so that a high level of Hct is maintained after returning to sea level. Villien *et al*.^10^ also observed that subjects still showed hypocapnia after returning to sea level.

The root cause of AMS associated headache remains unclear, largely due to the limitations of experimental tools and the environment required. This study used the state-of-art measure of CBF and revealed the different forms of CBF regulation between AMS and non-AMS subjects. This observation may be related to the “mismatch phenomenon” postulated by Wilson, *et al*.^38^ that links the headache to the impaired balance of cerebral venous drainage and arterial inflow. At sea level, restricted venous drainage with increased CBF would lead to venous engorgement and an increase in intracranial pressure (ICP), and subsequently to headache; the headache score was highly correlated with the level of venous engorgement^38^.

In the present study, we assumed that all subjects had a similar ability for venous drainage, and that increased CBF could result in increased ICP in AMS subjects which led to headache, while no evident CBF changes in non-AMS subjects would contribute to maintain the normal ICP.

Furthermore, the symptoms vanished and the LLS score decreased (<1) in 7 AMS subjects accompanied by a decline in CBF on day 6 at high altitude (HA2). With the CBF returning to the sea level value in AMS subjects, the ICP may also return to the normal range, which might explain the disappearance of AMS symptoms.

The main limitation of this study is the small sample size, especially in the non-AMS group. However, a larger sample size is logistically challenging in practice, and the non-AMS subjects can only be identified after arrival at altitude. Similar sample sizes were used in previous studies^10^,^16^,^17^,^18^,^39^. Another potential limitation of this study was the demographic distribution of the participants, only young and healthy adults with no previous exposure to hypoxia at high altitude were recruited, and that may affect the generalizability of the results. Nevertheless, the trend of the CBF change in the limited non-AMS participants was rather obvious and serves as convincing indicator for the different CBF regularization behaviours.

## Materials and Methods

### Subjects

Ten healthy young subjects (5 males and 5 females, 25.7 ± 1.6 years, body mass index 22.7 ± 2.7) were recruited for this study with written informed consent obtained. Participants were non-smokers, physically fit, taking no medication, living at 20–60 m above sea level and with no previous exposure to high altitude (>1500 m). No alcohol, caffeine, or medication that could affect CBF was consumed during this study. None of the subjects received medication to prevent AMS. The study was approved by the Ethics Committee of Chinese PLA General Hospital (registration number: S2015-014-02) and conformed to standards set by the Declaration of Helsinki.

### Experimental design

The overall experimental design was as follows: On day 1, all subjects received clinical tests, haematology, and MR examinations at sea level (Beijing, 50 m) (SL1). On day 2, all subjects flew from Beijing to Lhasa (3658 m) where they stayed for 5 days (day 2 to day 6). To ensure the timely measurement of CBF with acute exposure to hypoxia, MR examinations, including ASL and MRA, were performed within 6 h ± 2.5 h on average after arriving at high altitude (HA1) for all ten subjects. On day 6, MR examinations and haematology tests were repeated (HA2). The clinical tests were performed daily during the stay at high altitude. All subjects left Lhasa by train on day 6 and arrived at Beijing on day 8. Clinical tests and another MR examination were performed at sea level (SL2) on day 8. For logistical reasons, the cohort of 10 was divided into three sub-groups; the identical experimental procedure was followed for each sub-group. All experiments were completed within six weeks.

### Clinical tests and haematology

Blood pressure was measured using the Riva–Rocci method on the right arm under resting conditions. Systolic blood pressure (SP) and diastolic blood pressure (DP) were recorded, and MAP was calculated using the formula: MAP = (SP + 2 × DP)/3. SaO_2_ and HR were measured using an infrared fingertip pulse oximeter (OxyWatch C20, ChoiceMMed, USA) on the right index finger after 30 s of signal stabilization. Hct and Hb were measured from the peripheral venous samples using an automated haematology analyser (SE 9500, Sysmex Corporation, Kobe, Japan). Every morning at 8 am during the stay at high altitude, subjects were asked to complete the Lake Louise Score (LLS, 5 items) questionnaire^40^ to determine their status as AMS or non-AMS, a score of LLS >3 was considered to indicate AMS.

### MRI examination

Identical 3.0 T MR scanners (Discovery MR 750, GE Healthcare, Milwaukee, WI, USA) with an 8-channel head coil (*in vivo*) were used at both sea level and high altitude. The MR protocol included 3D pCASL, T1-weighted anatomical images, and 3D time of flight (TOF) magnetic resonance angiography (MRA). The 3D pCASL images were acquired with different PLD times of 1.5 s, 2.0 s, and 2.5 s. The scan parameters were as follows: 512 sampling points on eight spirals, spatial resolution = 3.64 mm, repetition time (TR) = 4632 ms (PLD = 1.5 s)/4844 ms (PLD = 2.0 s)/5327 ms (PLD = 2.5 s), echo time (TE) = 10.5 ms, bandwidth = ± 62.5 kHz, slice thickness = 4 mm, number of slices = 36, field of view (FOV) = 24 cm, number of excitations (NEX) = 3, acquisition time = 4 min 29 s (PLD = 1.5 s)/4 min 41 s (PLD = 2.0 s)/5 min 09 s (PLD = 2.5 s). A high resolution T1-weighted imaging dataset using a 3D fast spoiled gradient recalled echo (FSPGR) pulse sequence was acquired and used to generate a mask to separate grey matter and white matter. The parameters were as follows: TR = 6.9 ms, TE = 3.0 ms, TI = 450 ms, bandwidth = ± 31.25 kHz, FOV = 25.6 cm, slice thickness = 1 mm, matrix = 256 × 256, NEX = 1, acquisition time = 4 min 47 s. 3D TOF MRA was performed to measure the cross-sectional areas of major cerebral arteries with the following parameters: TR = 11 ms, TE = 2.4 ms, flip angle (FA) = 20, matrix = 256 × 256, NEX = 1, acquisition time = 2 min 54 s.

### Data processing

3D pCASL data were processed using ASL toolbox (http://www.fundacioncien.es/areas/asl_toolbox.asp), SPM8 (http://www.fil.ion.ucl.ac.uk/spm/software/spm8/) running with FSL (http://fsl.fmrib.ox.ac.uk/fsl/fslwiki/) and Matlab (MathWorks Inc., Natick, MA, USA). Firstly, DICOM images were imported into SPM, then T1-weighted images were co-registered to 3D pCASL NIFTI data after resampling. In order to exclude the signal from non-brain tissue, CBF images were processed using the skull stripping BET algorithm from FSL. Next, the registration of CBF images was implemented using the segmentation transformation matrix generated from T1-weighted images and spatially normalized to the template of the Montreal Neurological Institute. Finally, these normalized CBF data were smoothed with a 4 mm (FWHM) Gaussian kernel. Image quality controls were performed after every step via visual inspection.

**Figure 5:**
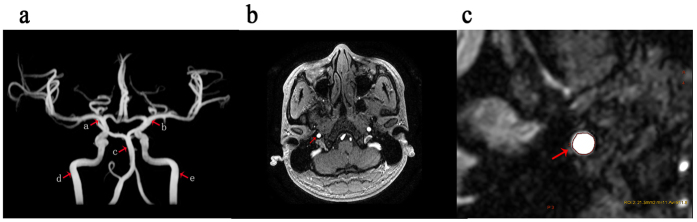
Images of the 3D time of flight MRA. (**a**) The sites where the cross-sectional area of ICA, BA and MCA were assessed. (**b**) Cross-sectional view of arrow d. (**c**) Calculation of the cross-sectional area.

To measure the cross-sectional areas of the ICA, BA, and MCA, the maximum intensity projection of TOF images were generated on a GE ADW4.5 workstation. An experienced neuroradiologist assessed the cross-sectional areas of bilateral ICAs, BA and bilateral MCAs, the measurements were averaged for bilateral vessels. The sites of measurement for the cross-sectional areas are illustrated in Figure 5. The cross-sectional areas of ICA were measured at the distal C1 segment of ICA, the cross-sectional areas of BA were measured at the site 10 mm away from the distal end of BA, and the cross-sectional areas of MCA were measured at the proximal M1 segment of MCA. The areas were measured by contouring the MIP arterial image and determined automatically by the workstation.

### Statistics

Statistical analysis was performed using SPSS software (version 17.0, SPSS, Chicago, IL, USA). Two-way classification ANOVA was conducted to compare differences among measurements obtained at different time points (SL1, HA1, HA2, and SL2). Comparisons between sea level (day 1) and high altitude (day 2) (HA1—SL1), day 2 and day 6 (HA2—HA1), before (day 1) and after (day 8) high altitude exposure (SL2—SL1) were made using paired samples *t*-test in all subjects and AMS subjects, while related samples nonparametric test was used for non-AMS subjects. Statistical analysis of AMS versus non-AMS subjects was performed using the independent sample nonparametric test. A P value of <0.05 was regarded as statistically significant. All values were shown as the mean ± sem.

## Additional Information

**How to cite this article**: Liu, W. *et al*. A longitudinal study of cerebral blood flow under hypoxia at high altitude using 3D pseudo-continuous arterial spin labeling. *Sci. Rep.* **7**, 43246; doi: 10.1038/srep43246 (2017).

**Publisher's note:** Springer Nature remains neutral with regard to jurisdictional claims in published maps and institutional affiliations.

## Figures and Tables

**Table 1:** Longitudinal changes in clinical data and haematology (mean ± sem).

		SL1	HA1	HA2	SL2	P-value
MAP (mmHg)	All	80.50 ± 3.20	79.00 ± 2.88	87.17 ± 1.99^#^	84.33 ± 1.63	0.015
	AMS	81.43 ± 3.63	91.90 ± 3.50	87.14 ± 1.98	84.29 ± 2.27	0.22
	non AMS	78.33 ± 7.64	72.22 ± 2.42	87.22 ± 9.77	84.44 ± 3.47	0.08
SaO_2_ (%)	All	98.40 ± 0.16	86.00 ± 1.24**	87.30 ± 0.96	98.30 ± 0.15	<0.001
	AMS	98.57 ± 0.20	86.14 ± 1.78**	87.29 ± 1.29	98.43 ± 0.20	<0.001
	non AMS	98.00 ± 0.00	85.67 ± 0.88	87.33 ± 1.45	98.00 ± 0.00	<0.001
HR (min^−1^)	All	69.30 ± 2.46	78.40 ± 3.85**	90.70 ± 4.04^##^	80.30 ± 3.33**	<0.001
	AMS	69.43 ± 3.46	80.43 ± 5.32*	90.57 ± 5.53^#^	81.14 ± 3.97**	0.004
	non AMS	69.00 ± 2.65	73.67 ± 2.73	91.00 ± 5.51	78.33 ± 7.31	0.059
Hct (L/L)	All	0.40 ± 0.01		0.44 ± 0.01^##^		<0.001
	AMS	0.40 ± 0.01		0.43 ± 0.01^##^		0.002
	non AMS	0.42 ± 0.02		0.47 ± 0.02		0.063
Hb (g/L)	All	136.40 ± 3.81		149.30 ± 4.38^##^		0.001
	AMS	134.86 ± 4.25		145.14 ± 5.12^##^		0.006
	non AMS	140.00 ± 9.07		159.00 ± 6.08		0.09

SL1: day 1, at sea level; HA1: day 2, at high altitude; HA2: day 6, at high altitude; SL2: day 8, returning to sea level; MAP: mean arterial pressure; SaO_2_: arterial oxygen saturation; HR: heart rate; Hct: hematocrit; Hb: Hemoglobin. *Significantly different compared to SL1; ^#^Significantly different compared to HA1.*P < 0.05;**P < 0.01; ^#^P < 0.05, ^##^P < 0.01.
